# Berberine Inhibits Nod-Like Receptor Family Pyrin Domain Containing 3 Inflammasome Activation and Pyroptosis in Nonalcoholic Steatohepatitis *via* the ROS/TXNIP Axis

**DOI:** 10.3389/fphar.2020.00185

**Published:** 2020-03-03

**Authors:** Weijian Mai, Yangzhi Xu, Jiahui Xu, Dan Zhao, Liangying Ye, Ganxiang Yu, Zhilei Wang, Qianting Lu, Jiaen Lin, Tao Yang, Chengxin Gu, Shiming Liu, Yun Zhong, Hui Yang

**Affiliations:** ^1^ Department of Gastroenterology, The Second Affiliated Hospital of Guangzhou Medical University, Guangzhou, China; ^2^ School of Clinical Pharmacy, Guangdong Pharmaceutical University, Guangzhou, China; ^3^ Guangzhou Institute of Cardiovascular Disease, The Second Affiliated Hospital of Guangzhou Medical University, Guangzhou, China

**Keywords:** berberine, non-alcoholic steatohepatitis, NLRP3 inflammasome, pyroptosis, reactive oxygen species

## Abstract

Berberine (BBR), an isoquinoline alkaloid originating from herbal plants, has been deemed beneficial for non-alcoholic fatty liver disease. Increasing evidence has demonstrated that Nod-like receptor family pyrin domain containing 3 (NLRP3) inflammasome activation and the subsequent pyroptosis contribute to the progression of non-alcoholic steatohepatitis (NASH). However, whether BBR impacts NLRP3 inflammasome activation and pyroptosis in NASH and the potential mechanism remains unclear. In the current study, we found that BBR significantly decreased lipid accumulation, ameliorated reactive oxygen species (ROS) and lipid peroxides, Tumor necrosis factor alpha (TNF-α) expression, and phosphorylation of Nuclear factor kappa B (NF-κB) p65 both *in vivo* and *in vitro.* In particular, BBR significantly inhibited NLRP3 expression, caspase-1 activity, and the pyroptosis executor, GSDMD-N, expression. In addition, BBR displayed similar inhibitory effects on NLRP3 inflammasome and pyroptosis with a decrease in ROS levels and TXNIP expression as N-acetyl-cysteine, a ROS scavenger, did. Whereas, the inhibitory effect of BBR on ROS, TXNIP expression, NLRP3 inflammasome activation and pyroptosis could be reversed by H_2_O_2_ in AML12 cells. This study demonstrates that BBR's inhibitory effect on NLRP3 inflammasome activation and pyroptosis may be mediated by ROS/TXNIP axis *in vitro* for the first time. Our findings suggest BBR is a potential candidate for the treatment of NASH.

## Introduction

Nonalcoholic fatty liver disease (NAFLD) includes two pathologically distinct conditions with different prognoses: non-alcoholic fatty liver (NAFL) and non-alcoholic steatohepatitis (NASH), which can eventually progress to end-stage liver diseases such as cirrhosis and hepatocellular carcinoma (Wong et al., 2014; Wong et al., 2016). Although the transition from NAFL to NASH has been considered the critical step in NAFLD development, understanding the mechanism and being able to clinically manage the disease require further investigations. Besides lifestyle changes, medications such as metformin, vitamin E, and Pioglitazone, are currently used to treat NAFLD in the clinic, but they often exert minor efficacy and considerable side effects (Wong et al., 2016; Sumida and Yoneda, 2018). Owing to these shortcomings, researchers are now focused on developing new therapeutics for NAFLD.

NLRP3 inflammasome complex, implicated in the progression of NAFLD from NAFL to NASH, contains NOD-like receptor pyrin domain containing 3 (NLRP3), a speck-like protein and damage-associated adaptor molecule with a caspase recruitment domain (ASC) and procaspase-1 as its effector molecule (He K. et al., 2017). NLRP3 inflammasome can be activated by many stimuli such as viruses, bacteria, free fatty acids (FFA), or reactive oxygen species (ROS). Once the complex is formed, inflammasomes activate caspase-1, which proteolytically activates the pro-inflammatory cytokines, interleukin-1β (IL-1β), and IL-18. Inflammasome activation also results in a rapid, pro-inflammatory form of cell death called pyroptosis (Latz et al., 2013).

Pyroptosis is a new form of programmed cell death resulting in cell swelling, membrane rupture, pore formation in the plasma membrane, extensive leakage of cytosolic contents, and high degree of inflammation (Shi et al., 2015; Shi et al., 2017). Pyroptosis is suggested to occur in cells (e.g., hepatocytes) where cytokine secretion is deemed infeasible following inflammasome-mediated caspase-1 activation (Miao et al., 2011). Gasdermin D (GSDMD), a major pyroptosis executor, exerts its function by releasing the N domain (GSDMD-N) cleaved by caspase 1/4/5/11. Recent studies have reported that GSDMD-mediated pyroptosis acts as an inflammatory link between NAFLD and NASH by controlling cytokine secretion, NF-ĸB activation, and lipogenesis (Shi et al., 2017; Xu et al., 2017).

Berberine (BBR), an isoquinoline alkaloid that originates from numerous herbal plants including *Coptis chinensis*, is used as a medicine for diarrhea and gut infections (Zhu et al., 2016). Recently, many other potential therapeutic effects of BBR for various diseases including central nervous system disorders, diabetes, cancer, cardiovascular disease, depression, hypertension, hypercholesterolemia, etc. have been explored (Kulkarni and Dhir, 2010; Vuddanda et al., 2010; Huang et al., 2018). BBR also displays a potent beneficial eﬀect in NAFLD by improving insulin resistance *via* multiple processes, reducing lipid accumulation *via* regulating AMPK signaling, improving mitochondrial function, alleviating oxidative stress, reducing serum cholesterol, and regulating gut microenvironment. Nonetheless, whether BBR impacts NLRP3 inflammasome activation, the subsequent pyroptosis in NASH, and the potential mechanism whereby these occur, remain unclear. Considering the importance of NLRP3 inflammasome and pyroptosis in NASH, the above activities require exploration.

Therefore, we examined the protective effects of BBR, particularly on NLRP3 inflammasome activation and pyroptosis, and explored the potential mechanism by which these occur. Our results showed that BBR inhibits NLRP3 inflammasome activation and the subsequent pyroptosis, and may be mediated by ROS/TXNIP axis in AML12 cells treated with methionine-choline deficient (MCD) medium plus lipopolysaccharide (LPS) or palmitic acid (PA).

## Materials and Methods

### Reagents and Antibodies

All reagents and chemicals were from Sigma-Aldrich (St. Louis, MO, United States) unless otherwise noted. Berberine was purchased from Selleck Chemicals (Houston, TX, United States. Cat. No. S2271), and the purity was 100%. Berberine was dissolved at a concentration of 80 mM in 100% DMSO as a stock solution, stored at −20°C and diluted with medium before each experiment. Nile red (Sigma-Aldrich, Cat. No. N3013) was dissolved at a concentration of 1 mg/ml in 100% DMSO as a stock solution, stored at −20°C and diluted with PBS before each experiment. DMEM/F-12 medium, MCD medium were purchased from Gibco (Waltham, MA, United States). The Prime Script RT Reagent Kit and SYBR Premix Ex Taq were purchased from TaKaRa (Dalian, China). N-acetyl-cysteine (NAC), DCFH-DA fluorescence probe, Lipid Peroxidation MDA Assay Kit, caspase-1 activity assay kit was purchased from Beyotime (Shanghai, China). Trizol, DNase I, radioimmunoprecipitation (RIPA) buffer, LipofectamineTM RNAiMAX transfection reagent were purchased from Invitrogen (Carlsbad, CA, United States). Fetal bovine serum (FBS), 1x insulin/transferrin/selenium (ITS) were purchased from SclenCell (Carlsbad, CA, United States). Rabbit anti-NLRP3 (#15101), rabbit anti-TXNIP (#14715), rabbit anti-NF-κB p65 (#4764S), rabbit anti-phospho-NF-κB p65 (#3033), mouse anti-β-actin (#3700), HRP-conjugated anti-rabbit (#7074), or anti-mouse IgG (#7076) antibodies were purchased from Cell Signaling Technology (Beverly, MA, United States). Rabbit anti GSDMD (ab209845), rabbit anti-TNF-α (ab215188), and rabbit anti-caspase-1 (ab179515) antibodies were purchased from Abcam (Cambridge, MA, United States).

### Cell Culture and Treatment

Immortalized mouse normal hepatocytes, AML12 cells were obtained from Stem Cell Bank, Chinese Academy of Sciences. AML12 cells were maintained at 37°C in DMEM/F-12 medium supplemented with 10% FBS, 1x ITS; and 40 ng/ml dexamethasone. At 70% confluence after serum starving for 12 h, cultured cells were treated with MCD medium for 24 h followed by a 12 h treatment with 1 μg/ml LPS or 1 mM PA for 24 h to establish the NASH cellular model and non-treated cells were used as control.

To assess the effect of BBR on MCD/LPS or PA-induced hepatocyte injury, two concentrations of BBR (10 or 20 μM) were added to the cells in combination with MCD/LPS or PA for 24 h. The selection of BBR concentration is based on our pilot experiment (**Figure S1**) and other published study (Guo et al., 2016). To assess the role of oxidative stress in BBR-mediated inhibition of NLRP3 inflammasome and pyroptosis *in vitro*, 10 μM NAC, a ROS scavenger, was applied in combination with MCD/LPS or PA for 24 h, and 200 nM H_2_O_2_, a ROS inducer, was applied in combination with BBR and MCD/LPS or PA for 6 h.

### Animal Experiments

Fifteen 7-week old male C57BLKS/J mice were obtained from Experimental Animal Center of Southern Medical University (Guangzhou, China). All mice were maintained on a 12/12 h light/dark cycle with free access to food and water. The mice were divided to the following three groups (5 mice per group): (i) control group, received a methionine and choline sufficient (MCS) diet (Dyets, USA, #519581) for 5 weeks and treated with PBS *via* oral gavages for the last 2 weeks; (ii) MCD group, fed a methionine and choline deficient (MCD) diet (Dyets, USA, #519580) for 5 weeks and treated with PBS *via* oral gavages for the last 2 weeks; and (iii) MCD + BBR group, received MCD diet for 5 weeks and treated with BBR (100 mg/kg body weight/d, suspension in PBS) *via* oral gavages for the last 2 weeks. At the age of 12 weeks, serum samples were collected prior to the sacrifice of mice and liver was harvested for further studies.

This study was approved by the ethical committee of Guangzhou Medical University, China. All the animal experiments complied with the standard ethical guidelines prescribed by the ethical committees mentioned above.

### Nile Red Staining

Intracellular lipids were stained with Nile Red (Sigma-Aldrich, cat. no. N3013) for 15 min after fixing with 4% paraformaldehyde for 30 min, and the nuclei stained with DAPI for an additional 5 min. Images were photographed with an inverted fluorescence microscope (EVOS FL, Thermo Fisher Scientific Inc., USA).

### Measurement of Intracellular ROS Level

For the quantitative analysis of ROS production, cells were incubated with DCFH-DA fluorescence probe for 30 min using the manufacturer's manual. Fluorescence intensity was measured by flow cytometry (FACS Calibur, BD Biosciences) and analyzed using FlowJo software version 10.0 (Treestar, Inc., Palo Alto, CA, USA).

### Measurement of Malondialdehyde

Measurement of malondialdehyde (MDA) levels in AML12 cells was performed using a Lipid Peroxidation MDA Assay Kit. MDA is an end-product of fatty acid peroxidation and can be measured *via* chromogenic reaction between MDA and Thiobarbituric acid (TBA). Briefly, 100 μl of cell lysate was mixed with 200 μl of MDA working solution followed by incubation in a 100°C water bath for 15 min, then cooled to 25°C. The mixture was centrifuged at 1,000 × g for 10 min, and 200 μl supernatant was used to measure absorbance at 532 nm. TBARS concentration was calculated from an MDA standard curve.

### Quantitative PCR

Total RNA from cultured cells was isolated using Trizol and treated with DNase I. In brief, quantitative real-time PCR was carried out to determine lncRNA expression using the Prime Script RT Reagent Kit and SYBR Premix Ex Taq. Real-time PCR was performed on ABI Prism 7300 real-time PCR system (Applied Biosystems, Foster City, CA, USA). Quantification of target gene expression was performed using the relative quantification comparative CT method. The primer sequences used for real-time PCR include: TNF-α, 5'-GCCACCACGCTCTTCTGTCTAC-3' (forward) and 5'-GGGTCTGGGCCATAGAACTGAT-3' (reverse); β-actin: 5'-ATGCCACAGGATTCCATACCCAAGA-3' (forward) and 5'-CTCTAGACTTCGAGCAGGAGATGG-3' (reverse).

### Assaying Caspase-1 Activity

Caspase-1 activity was detected using a caspase-1 activity assay kit according to the manufacturer's instructions. This assay was based on the ability of caspase-1 to convert acetyl-Tyr-Val-Ala-Asp p-nitroaniline (Ac-YVAD-pNA) to the yellow formazan product, p-nitroaniline (pNA). Thus, the activity of caspase-1 can be assessed by measuring the absorbance of pNA using a standard pNA curve. Fifty *μ*g of total cytosolic protein was incubated in a 96-well microtiter plate with 20 nmol Ac-YVAD-pNA overnight at 37°C. The absorbance values at 405 nm were measured using the spectrophotometer, Varioscan Flash, and SkanIt Software Version 2.4.3. RE (Thermo Fisher Scientific Inc, USA). The production of pNA in the tested samples indicated the level of caspase-1 activation.

### Histopathology Analysis

Liver tissue samples were fixed in 4% paraformaldehyde in phosphate-buffered saline (PBS) for 2 h, stored overnight in 10% formalin, and were embedded in paraffin. Cross-sections (5 µm) of the tissue were cut and used for staining with hematoxylin and eosin. To evaluate the degree of hepatic lipid accumulation, oil red O staining of the sections was performed using an oil red O staining kit (Nanjing Jiancheng Bioengineering Institute) following the manufacturer's instructions. Images were taken using a light microscope. The NAFLD activity score (NAS) were performed by an experienced pathologist without prior knowledge of the treatments. The scoring includes measurement of steatosis grade (0-no steatosis, 1- < 50% steatosis, 2-> 50% steatosis, 3- > 50% steatosis + microvesicular steatosis), hepatocyte ballooning (0-none, 1- < 66%, 2- > 66% hepatocyte involvement) and inflammation (0-no foci, 1- < 2 foci, 2–2–4 foci, 3-+4 foci). NASH was defined in the cases of NAS of ≥5 (Kleiner et al., 2005; Zhang et al., 2016).

### Measurement of ALT and AST

After centrifuged at 6,000 rpm for 10 min, serum levels of aspartate aminotransferase (AST) and alanine aminotransferase (ALT) were then measured using Olympus AU5400 clinical biochemical analyzer (Tokyo, Japan).

### Western Blot

Western blot was performed as previously described (Chen et al., 2017). For total cellular protein extraction, cells were lysed in 1X radioimmunoprecipitation (RIPA) buffer supplemented with protease and phosphatase inhibitors (Roche Applied Science, IN, USA). Equal amounts of proteins were loaded on 7.5–12.5% sodium dodecyl sulfate polyacrylamide gel electrophoresis (SDS-PAGE) and transferred to a nitrocellulose transfer membrane (Bio-Rad, CA, USA). The membrane was then blocked by PBS supplemented with 0.1% Tween 20 and 5% non-fat dry milk (PBST-milk) for 1 h at room temperature. Overnight incubation with the following primary antibodies was performed at 4°C: rabbit anti-NLRP3 (1:1,000), rabbit anti GSDMD (1:1,000), rabbit anti-TXNIP (1:1,000), rabbit anti-TNF-α (1:1,000), rabbit anti-NF-κB p65 (1:1,000), rabbit anti-phospho-NF-κB p65 (1:1,000), rabbit anti-caspase-1 (1:1,000), and mouse anti-β-actin (1:5000). After three 5-min washes in PBST, membranes were incubated with HRP-conjugated anti-rabbit 1:5,000) or anti-mouse IgG 1:5000) for 1 h. Blots were developed using ECL chemiluminescence reagent (Super Signal West Pico Chemiluminescent Substrate; Pierce; Thermo Fisher Scientific, Inc.). The results of Western blot represent at least three independent experiments. β-actin was used as a loading control.

The intensity of each band was quantified using ImageJ software and normalized to β-actin and adjusted relative to the MCD/LPS or PA treated group.

### Statistical Analysis

Results are presented as means ± standard deviation. Data were analyzed using the Student's t-test (for two groups) or one-way ANOVA and Fish's Least Significant Difference (LSD) test (for more than two groups). P values < 0.05 were considered statistically significant. All analyses were performed using the Statistical Package for Social Sciences, version 20.0, for Windows (SPSS, Inc., Chicago, IL, USA).

## Results

### Berberine Decreases PA or MCD Medium-Induced Lipid Accumulation in AML12 Cells

**Figure 1:**
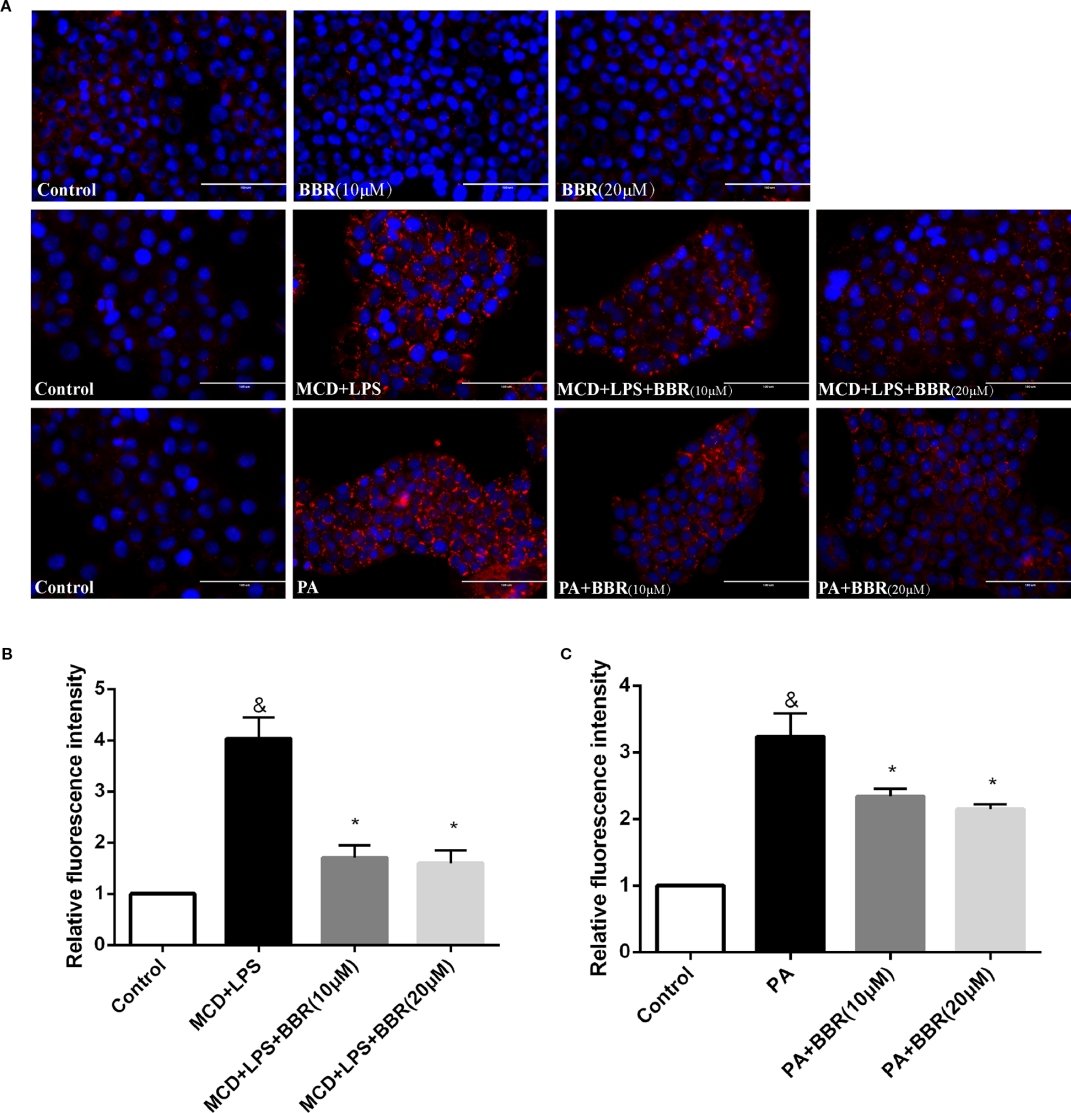
Berberine (BBR) decreased methionine-choline deficient (MCD)/lipopolysaccharide (LPS) and PA-induced lipid accumulation in AML12 cells. **(A)** Representative images of Nile red staining of AML12 cells. AML12 cells received MCD medium treatment for 24 h followed by a 12-h LPS treatment or 24-h PA treatment, respectively, to establish non-alcoholic steatohepatitis (NASH) *in vitro* model. Two concentrations of BBR (10 or 20 μM) were added to the cells alone or in combination with MCD/LPS or PA for 24 h. Intracellular lipid accumulation was assessed by Nile red staining. **(B, C)** The relative Nile red fluorescence intensity of each group was measured using flow cytometry. Data were expressed as mean ± SD of three independent experiments. &p < 0.05 vs control group, *p < 0.05 vs MCD+LPS or PA group. Scale bar, 100 μM. MCD, Methionine-choline deficient; LPS, lipopolysaccharide; PA, palmitic acid; NASH, non-alcoholic steatohepatitis.

As lipid accumulation is a key feature of NAFLD, we established two NASH *in vitro* models to mimic hepatic steatosis and injury in NASH. AML12 cells were exposed to a 24-h MCD-medium treatment plus a 12 h LPS (1 μM) treatment or 24 h PA (1 mM) treatment and non-treated cells were used as control. Evidently, there was lipid accumulation in the MCD/LPS or PA treatment groups compared to the non-treated group by Nile red staining. We then explored the effect of BBR on lipid accumulation in AML12 cells by administering two doses (10 and 20 μM) in the presence or absence of MCD/LPS or PA. Both doses significantly and dose-dependently decreased lipid accumulation in AML12 cells in the presence of MCD medium/LPS or PA, as shown by Nile red staining and flow cytometric analysis (**Figure 1**).

### Oxidative Stress in PA or MCD-Treated AML12 Cells is Ameliorated by BBR

**Figure 2:**
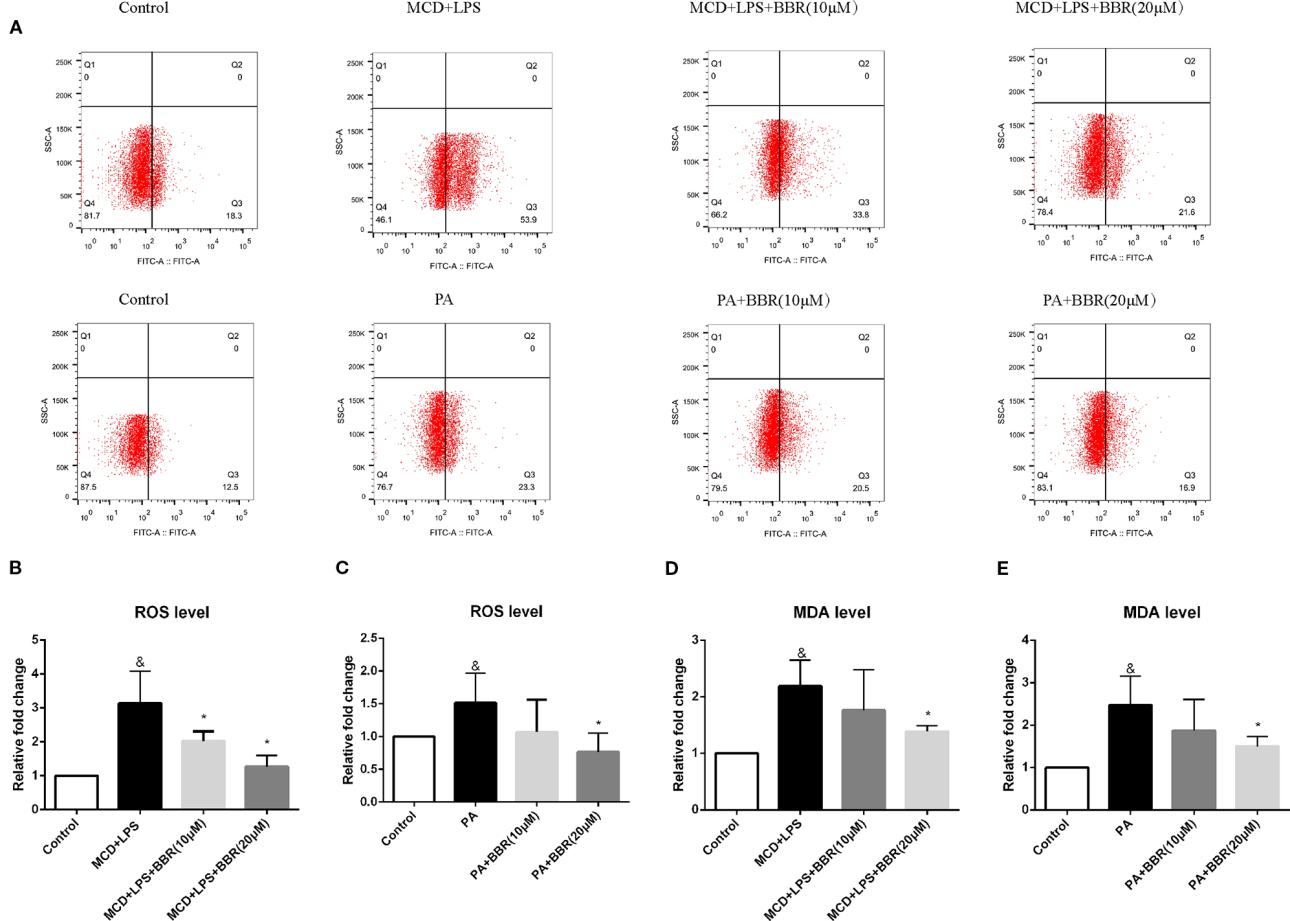
The effect of berberine (BBR) on oxidative stress in PA or methionine-choline deficient (MCD)/lipopolysaccharide (LPS)-treated AML12 cells. AML12 cells were treated with MCD/LPS or PA in the presence or absence of BBR at two concentrations for 24 h. **(A)** Representative images of flow cytometry analysis for intracellular reactive oxygen species (ROS) production. Effects of BBR on ROS levels were assessed by flow cytometry using DCF-DA assay. The lower-right quadrants of each plot indicate the percentage of ROS+ cells. **(B, C)** The proportion of cells producing ROS detected by flow cytometry using DCF-DA assay was compared to that observed in the control group. **(D, E)** The relative MDA levels were measured by MDA Assay Kit. Data were expressed as mean ± SD of three independent experiments. &p < 0.05 vs control group, *p < 0.05 vs MCD+LPS or PA group. MCD, Methionine-choline deficient; LPS, lipopolysaccharide; PA, palmitic acid; ROS, reactive oxygen species.

As oxidative stress contributes to the initiation and progression of liver injury in NAFLD, we determined the antioxidative effect of BBR by assessing intracellular ROS using a DCF assay. **Figure 2A** shows representative results of flow cytometric analysis of ROS levels in AML12 cells treated with MCD/LPS or PA in the presence or absence of 10 or 20 μM BBR. **Figures 2B, C** indicate the proportion of cells producing ROS in each group for three independent experiments. Compared to the control group, the percentage of cells producing ROS was higher in the NASH cellular model groups, indicating increased ROS levels in MCD/LPS-treated or PA-treated AML12 cells. Administering 10 μM BBR in the presence of MCD/LPS and 20 μM BBR in the presence of MCD/LPS and PA significantly attenuated ROS levels in AML12 cells.

To further verify the anti-oxidative effect of BBR, we assessed the level of the lipid peroxidation product, MDA, in BBR-treated AML12 cells with an MDA assay kit. Similar to the intracellular ROS levels, MDA concentration was increased in the MCD/LPS group and PA group, and reduced in the presence of MCD/LPS after 10 and 20 μM BBR treatment and in the presence of PA after 20 μM BBR as shown in **Figures 2D, E**.

### Berberine Inhibits NFκB p65 Phosphorylation and TNF*α* Expression in AML12 Cells

**Figure 3:**
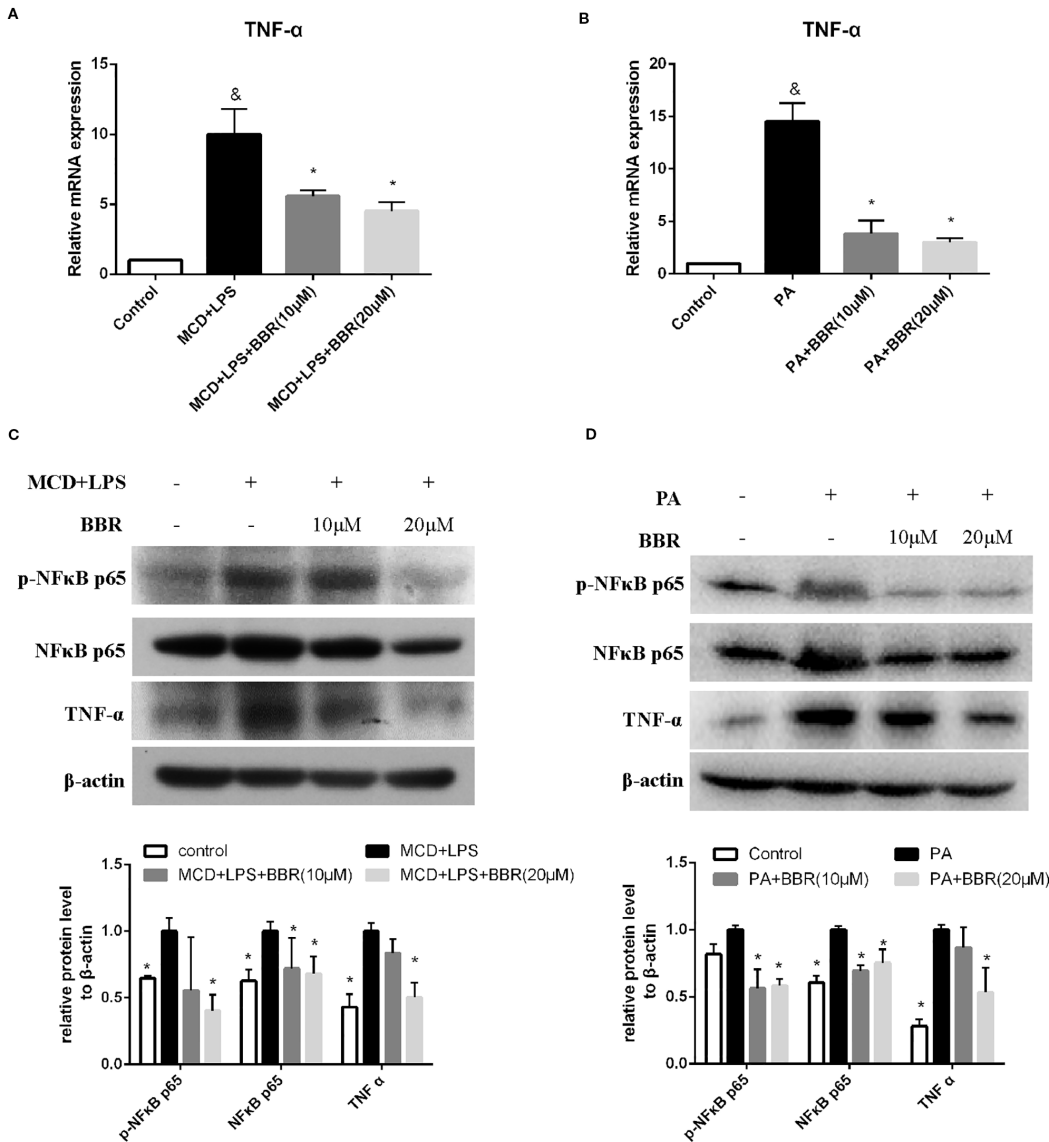
Berberine (BBR) inhibited NF-κB p65 phosphorylation and TNFα expression in AML12 cells. AML12 cells were exposed to methionine-choline deficient (MCD)/lipopolysaccharide (LPS) or PA in the presence or absence of BBR at two concentrations for 24 h. **(A, B)** The mRNA expressions of TNF-α are assessed by qPCR. **(C, D)** The phosphorylation of NF-κB p65, and the protein levels of NF-κB p65 and TNFα were assessed by immunoblotting. Data were expressed as mean ± SD of three independent experiments. ^&^p < 0.05 vs control group, *p < 0.05 vs MCD/LPS or PA group. MCD, Methionine-choline deficient; LPS, lipopolysaccharide; PA, palmitic acid.

TNF-α and NF-κB signaling play pivotal roles in the development and progression of NAFLD. Phosphorylation of p65 at Ser 536 leads to nuclear localization of the transcriptionally active complex and NF-kB mediated transactivation of several downstream genes. Thus, we examined the effect of BBR on TNF-α expression and NF-κB signaling by qRT-PCR and western blot, respectively. As shown in **Figure 3A**, qRT-PCR results revealed that TNF-α mRNA expression was significantly increased in the MCD/LPS group compared to the control group, and MCD/LPS-induced elevation was downregulated due to BBR in AML12 cells. Western blot showed that both TNF-α and phosphorylation of NF-κB p65 were increased by MCD/LPS treatment compared to the controls; these were inhibited by BBR, particularly the 20 μM dose (**Figure 3C**). Consistent with the results obtained in MCD/LPS-treated AML12 cells, mRNA and protein expression of TNF-α, as well as the phosphorylation level of NF-κB p65 were lower in the BBR treatment group compared to the PA treatment group (**Figures 3B, D**).

### Berberine Suppresses NLRP3 Inflammasome Activation and Pyroptosis *In Vitro*

**Figure 4:**
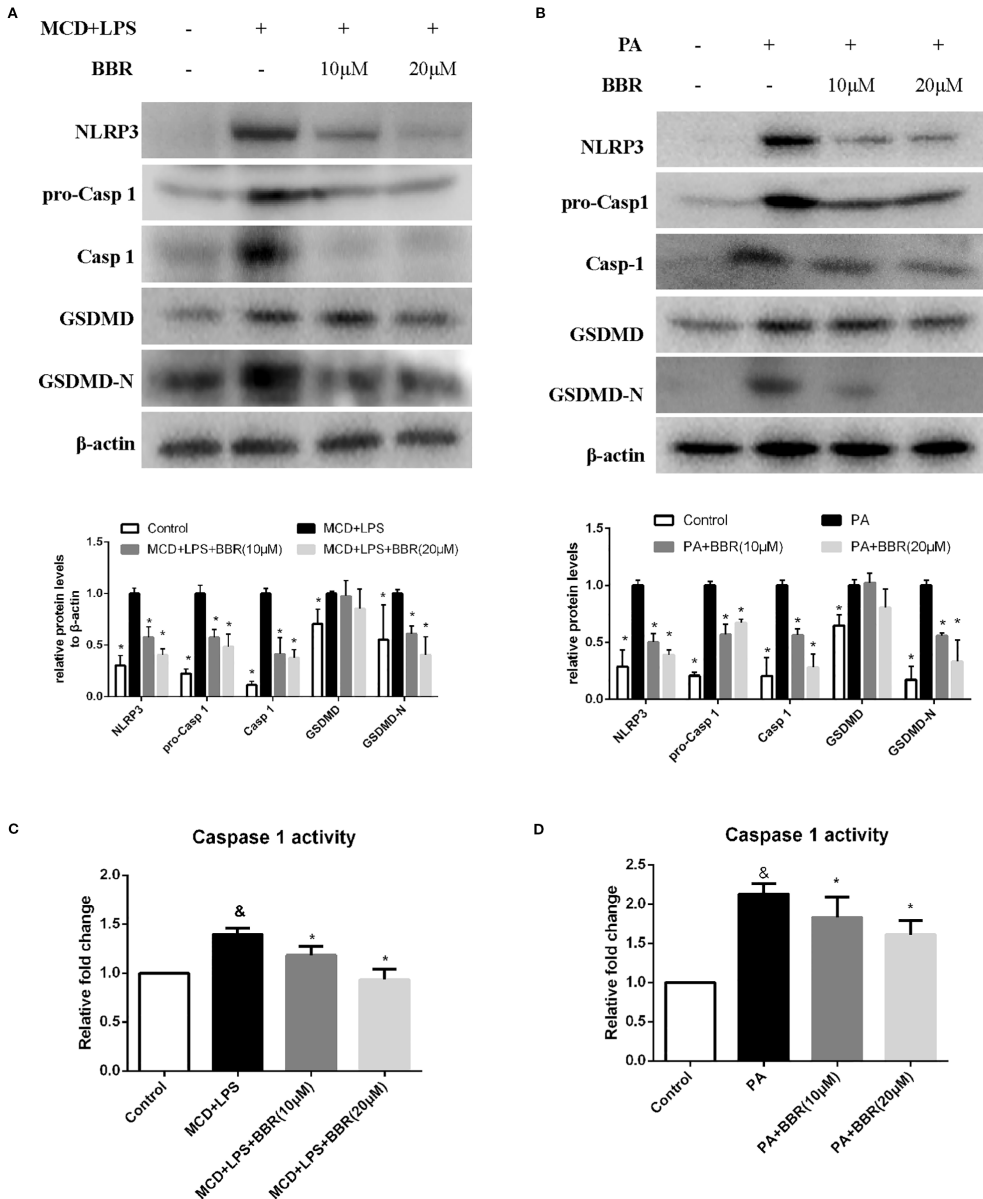
Berberine (BBR) suppressed NLRP3 inflammasome activation and pyroptosis. AML12 cells were exposed to methionine-choline deficient (MCD)/lipopolysaccharide (LPS) or PA in the presence or absence of BBR at two concentrations for 24 h. **(A, B)** Western blot of the protein levels of NLRP3, pro-caspase 1, caspase 1, GSDMD and GSDMD-N in AML12 cells. **(C, D)** Caspase-1 activity was determined using a caspase-1 activity assay. Data were expressed as mean ± SD of three independent experiments. ^&^p < 0.05 vs control group, *p < 0.05 vs MCD+LPS or PA group. MCD, Methionine-choline deficient; LPS, lipopolysaccharide; PA, palmitic acid; GSDMD, gasdermin D.

NLRP3 inflammasome activation may lead to GSDMD-driven pyroptosis, which plays a key role in the pathogenesis of NASH (Wree et al., 2014a). We aimed to determine whether BBR could attenuate NLRP3 inflammasome activation and the subsequent pyroptosis. As shown in **Figure 4**, we found that both MCD/LPS and PA treatment significantly increased NLRP3, pro-caspase 1 and caspase1 protein levels, and caspase-1 activity, indicating NLRP3 inflammasome activation. Moreover, GSDMD and its cleaved N domain (GSDMD-N), the pyroptosis executor, were also increased in the MCD/LPS and PA treatment groups. BBR treatment, however, dose-dependently ameliorated MCD/LPS or PA-induced activation of NLRP3 inflammasome and pyroptosis as shown by the decreased expression of NLRP3, pro-caspase 1 and caspase1, GSDMD-N protein, and capspase-1 activity. As the 20 μM concentration of BBR displayed more obvious protective effects on NASH, this was selected for use in the subsequent experiments.

### Berberine Suppresses NLRP3 Inflammasome Activation and Pyroptosis *via* the ROS/TXNIP Axis

**Figure 5:**
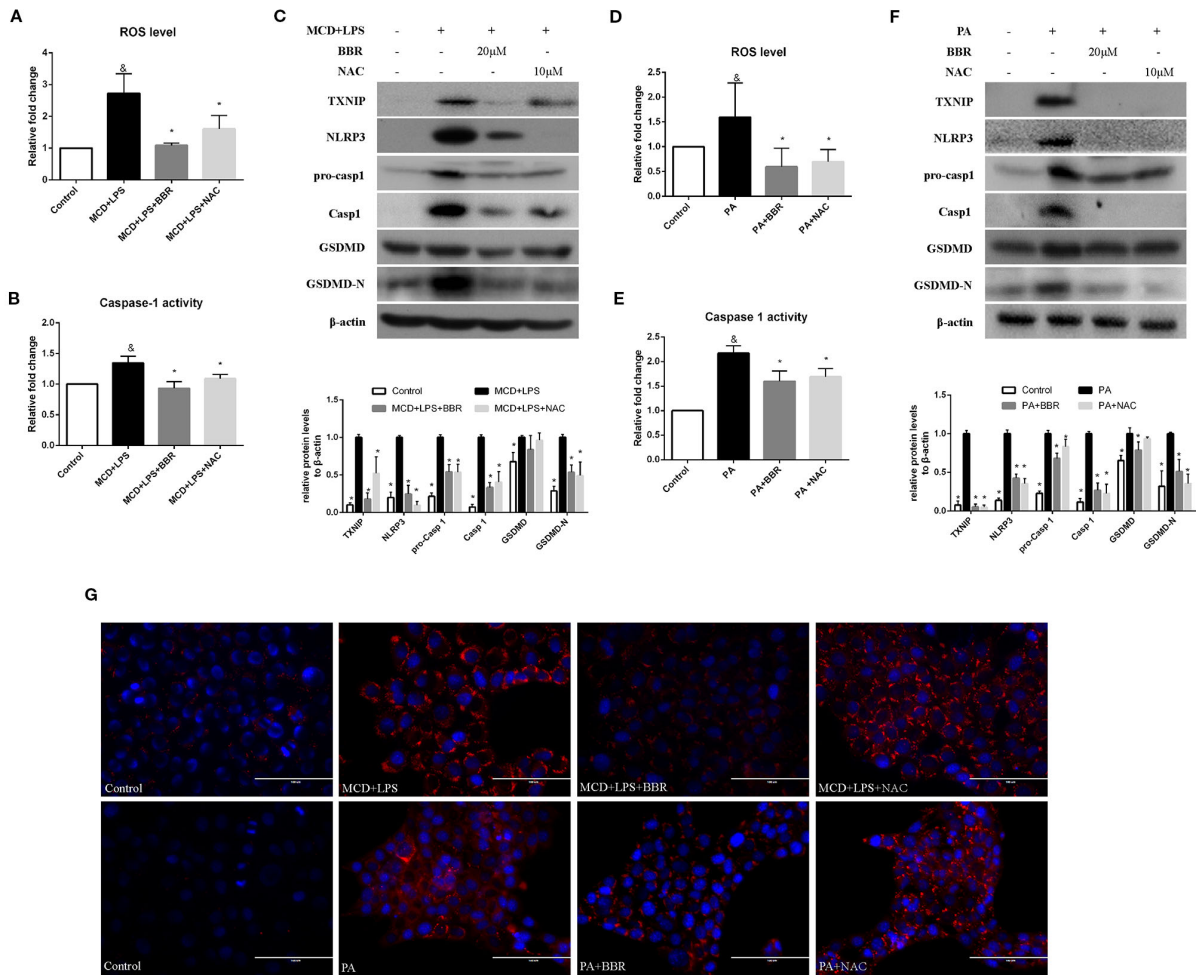
Comparison of the effects of BBR and NAC on oxidative stress, NLRP3 inflammasome activation, pyroptosis and lipid accumulation in AML12 cells. AML12 cells were exposed to MCD/LPS or PA in the presence or absence of 20 μM BBR or 10 μM NAC for 24 h. **(A, D)** The proportion of cells producing reactive oxygen species (ROS) detected by flow cytometry using DCF-DA assay was compared to control group. **(B, E)** Caspase-1 activity was determined using a caspase-1 activity assay. **(C, F)** Western blot for TXNIP, NLRP3, pro-caspase 1, caspase 1, GSDMD, and GSDMD-N in AML12 cells. **(G)** Representative images of Nile red staining of AML12 cells. Data were expressed as mean ± SD of three independent experiments. ^&^p < 0.05 vs control group, *p < 0.05 vs MCD+LPS or PA group. MCD, Methionine-choline deficient; LPS, lipopolysaccharide; PA, palmitic acid; GSDMD, gasdermin D; NAC, N-acetyl-cysteine.

It has been reported that ROS can trigger TXNIP to dissociate from TRX and bind to NLRP3, resulting in NLRP3 activation (Zhou et al., 2010). Based on the above findings, we speculated that BBR may inhibit NLRP3 inflammasome and pyroptosis in NASH *via* the ROX-TXNIP axis. To explore the potential underlying mechanism, NAC, a ROS scavenger, was introduced into the experiment and the inhibitory effect of BBR and NAC on oxidative stress, NLRP3 inflammasome activation, cellular pyroptosis, and lipid accumulation was compared. As shown in **Figure 5**, BBR (20 μM) and NAC (10 μM) decreased the cellular ROS levels in MCD/LPS or PA-treated AML12 cells to a similar degree. As expected, TXNIP, NLRP3, pro-caspase 1, caspase1, and GSDMD-N protein levels and caspase-1 activity were suppressed by NAC, a result similar to BBR. However, as shown by Nile red staining, NAC failed to alleviate MCD/LPS or PA-induced lipid accumulation as BBR. These results indicate that the ROS-TXNIP axis may be involved in BBR's inhibitory effect on NLRP3 inflammasome and the subsequent cellular pyroptosis.

**Figure 6:**
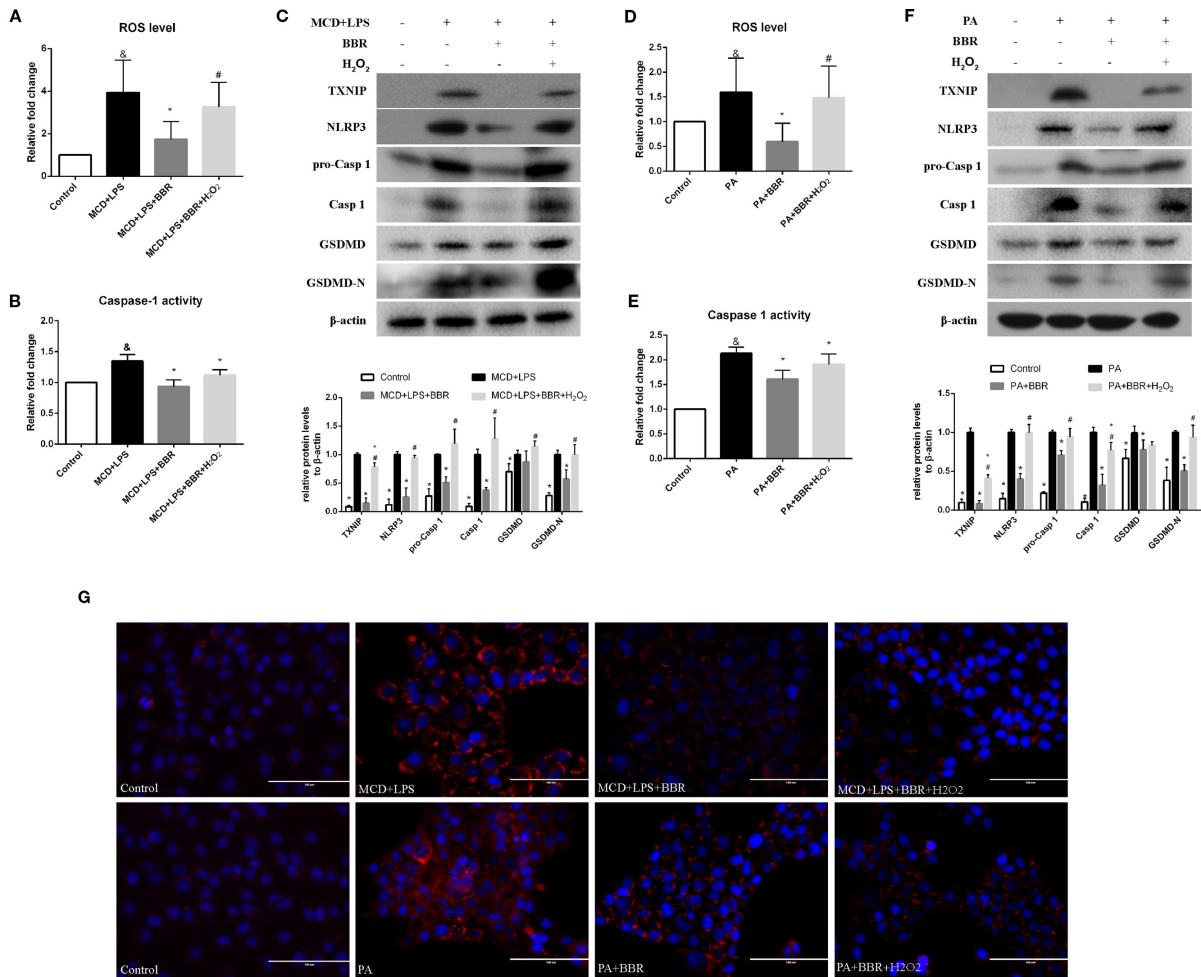
The inhibitory effect of berberine (BBR) on nod-like receptor family pyrin domain containing 3 (NLRP3) inflammasome activation and pyroptosis was reversed by H_2_O_2_. AML12 cells were exposed to methionine-choline deficient (MCD)/lipopolysaccharide (LPS) or PA in the presence or absence of 20 μM BBR for 24 h. H_2_O_2_ (200 μM) was added to cells in the final 6 h in the indicated group. **(A, D)** The proportion of cells producing reactive oxygen species (ROS) detected by flow cytometry using DCF-DA assay was compared to control group. **(B, E)** Caspase-1 activity was determined using a caspase-1 activity assay. **(C, F)** Western blot for TXNIP, NLRP3, pro-caspase 1, caspase 1, GSDMD and GSDMD-N in AML12 cells. **(G)** Representative images of Nile red staining of AML12 cells. Data were expressed as mean ± SD of three independent experiments. ^&^p < 0.05 vs control group, *p < 0.05 vs MCD+LPS or PA group, ^#^p < 0.05 vs MCD+LPS+BBR or PA+BBR group. MCD, Methionine-choline deficient; LPS, lipopolysaccharide; PA, palmitic acid; ROS, reactive oxygen species; GSDMD, gasdermin D.

To further confirm these findings, H_2_O_2_, a ROS inducer, was administered to the BBR treatment group by exposing the AML12 cells to H_2_O_2_ for 6 h. We observed that there was no lowering in cellular ROS levels in this group compared to the MCD/LPS or PA group, indicating the abolition of the anti-oxidative effect of BBR (**Figures 6A, D**). Western blot showed that the decreases in TXNIP, NLRP3, pro-caspase 1, caspase1, GSDMD-N protein levels, and caspase-1 activity caused by BBR in MCD/LPS or PA-treated AML12 cells were reversed, and the activation of NLRP3 inflammasome and pyroptosis was rescued to varying degrees by H_2_O_2_ (**Figure 6**). While, Nile red staining showed that H_2_O_2_ administration fail to reverse the BBR-induced amelioration of lipid accumulation, indicating that ROS may not involve in BBR-mediated improvement of lipid accumulation in NASH (**Figure 6G**). Taken together, these data reveal that the inhibitory effect of BBR on NLRP3 inflammasome and the subsequent cellular pyroptosis in MCD/LPS or PA-treated AML12 cells may be mediated by the ROS/TXNIP axis.

### Berberine Ameliorated Steatohepatitis in MCD-Fed Mice

**Figure 7:**
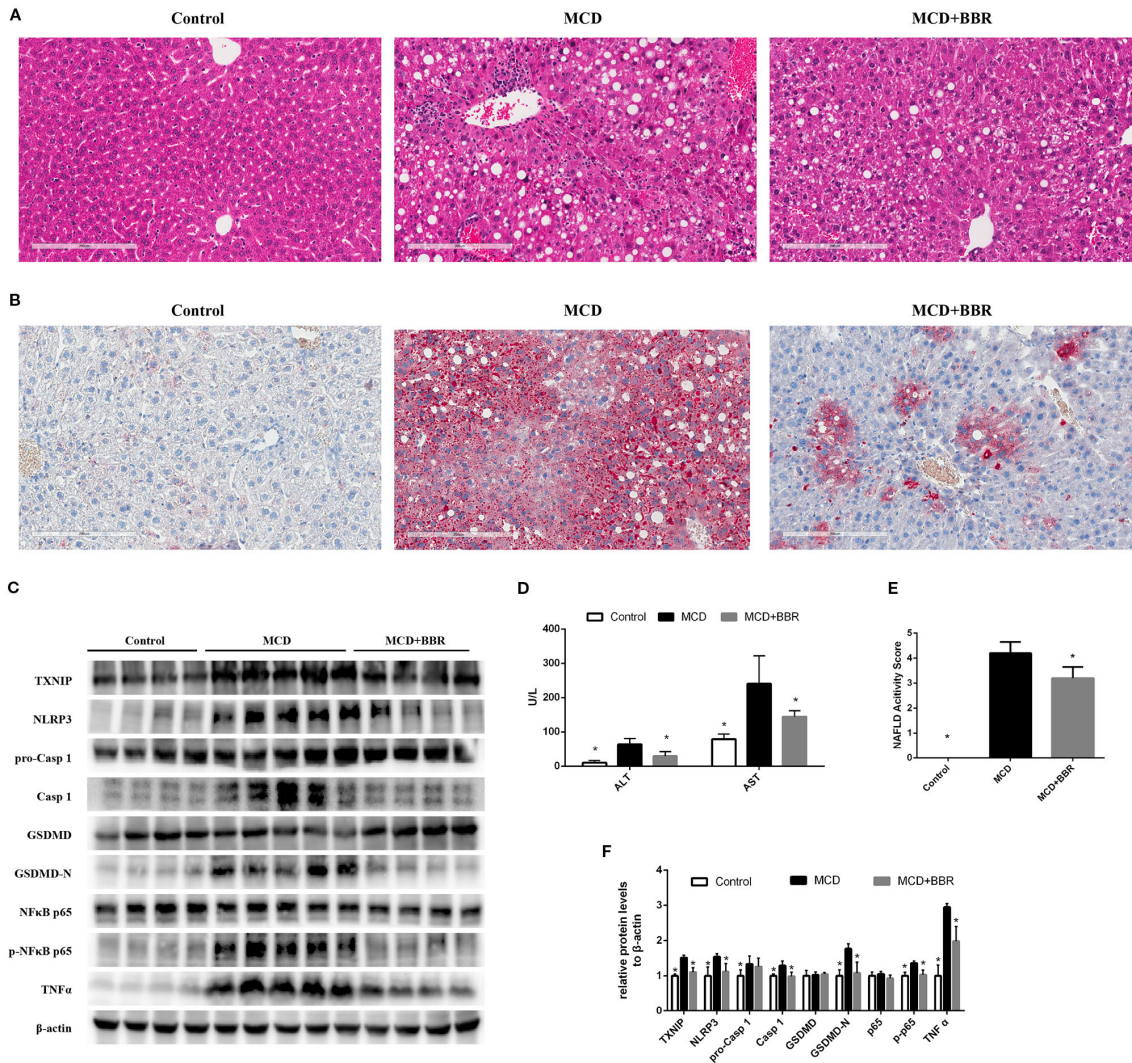
Berberine (BRB) treatment ameliorated hepatic steatosis, liver injury and pyroptosis in methionine-choline deficient (MCD)-fed mice. Male C57BL/6J mice, received MCD diet for 5 weeks and treated with BBR (100 mg/kg body weight/d, suspension in PBS) *via* oral gavages for the last 2 weeks. **(A)** Representative images of the Hematoxylin and Eosin (H&E) and **(B)** oil red O stained liver of mice. **(C, F)** Western blot for the indicated proteins in mice liver. **(D)** Serum ALT and AST level of mice. **(E)** NAFLD activity score of mice. Data were expressed as mean ± SD. *p < 0.05 vs MCD group. ALT, Alanine aminotransferase; AST, Aspartate aminotransferase; MCD, Methionine-choline deficient; GSDMD, gasdermin D.

To validate the findings in AML12 cells, we examined the protective effect of BBR in MCD-induced steatohepatitis mice. As shown in **Figure 7**, upon feeding MCD diet for 5 weeks, lipid droplets and inﬂammatory foci within the lobule in MCD-fed mice were observed by HE staining (**Figure 7A**). Oil red O staining of the liver sections further confirmed the fat accumulation in MCD-fed mice (**Figure 7B**). Liver injury was occurred as reflected in the elevation of ALT and AST (**Figure 7D**). These results indicated steatohepatitis was developed in MCD-fed mice. However, 2-week BBR treatment significantly ameliorated hepatic steatosis and liver injury induced by MCD diet. And the NAS system also revealed that the liver histology in the BBR-administered group was improved (**Figure 1E**).

Furthermore, Western blot showed that the level of TXNIP, NLRP3 inflammasome, pyroptosis, phosphor p65, and TNFα was increased in the liver of MCD group. Whereas 2-week BBR treatment to MCD-fed mice significantly decreased the expression of TXNIP, NLRP3, caspase-1, GSDMD-N, phosphor p65, and TNFα (**Figures 7C, F**), which was consistent with the findings in AML12 cells. Taken together, our *in vivo* study indicated that BBR may ameliorated steatohepatitis in MCD-fed mice.

## Discussion

**Figure 8:**
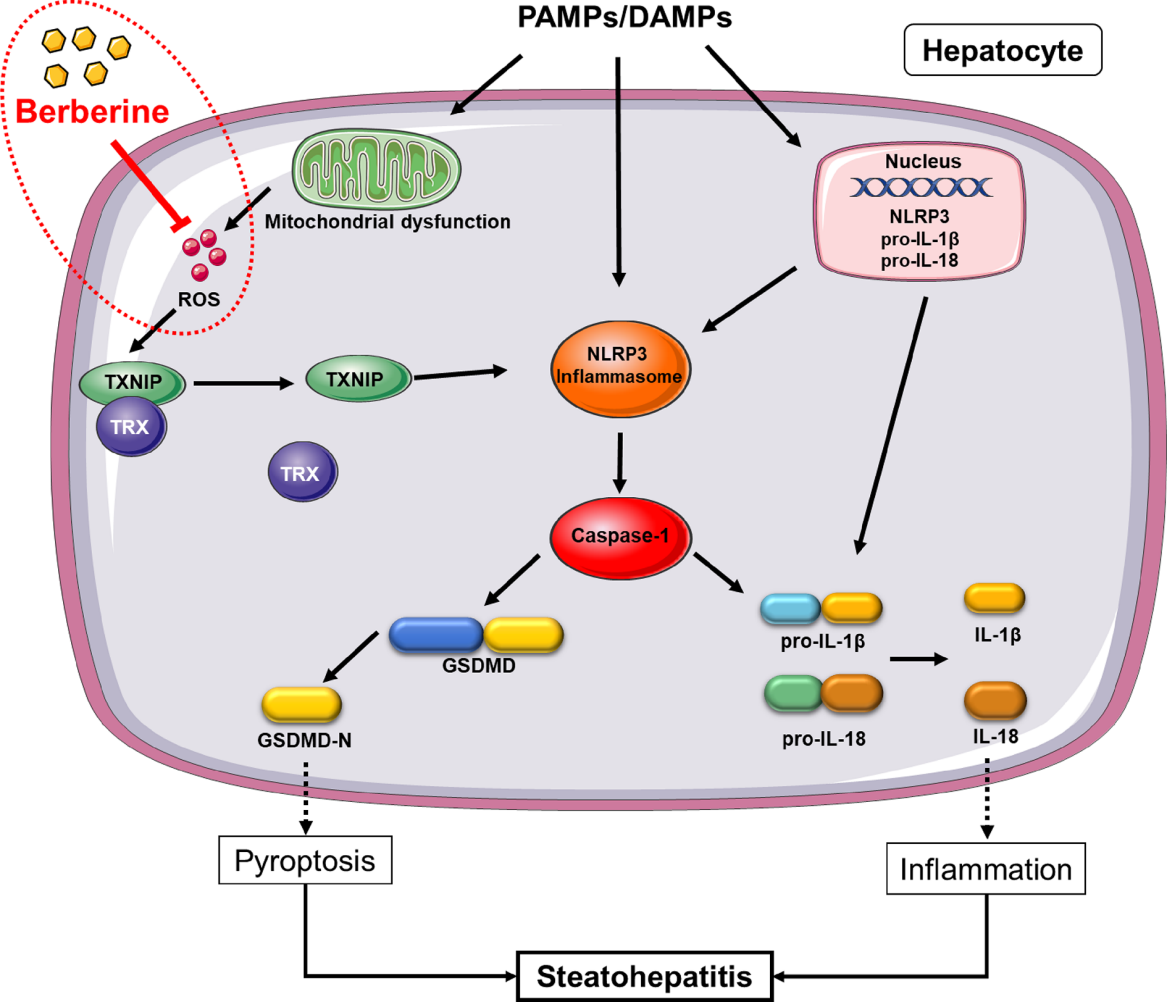
Proposed mechanism of berberine (BBR) inhibiting reactive oxygen species (ROS)/nod-like receptor family pyrin domain containing 3 (NLRP3)/pyroptosis signaling. In non-alcoholic steatohepatitis (NASH), NLRP3 inflammasome can be activated by a large number of stimuli including PA, LPS, etc. *via* priming and activation signaling, to result in caspase-1 activation. Caspase-1 contributes to the maturation and secretion of IL-1β and IL-18, and cleaves GSDMD which results in plasma membrane pore formation and pyroptosis. ROS, an activation signal, trigger TXNIP to dissociate from TRX and bind to NLRP3, leading to NLRP3 activation. Berberine inhibits NLRP3 inflammasome and pyroptosis by decreasing ROS generation. PAMPs, pathogen associated molecular patterns; DAMPs, damage associated molecular patterns; ROS, reactive oxygen species; GSDMD, gasdermin D; PA, palmitic acid; LPS, lipopolysaccharides; NASH, non-alcoholic steatohepatitis.

Although many researched have been performed to explore the therapeutic effect and mechanism of BBR in diseases, its effect on NLRP3 inflammasome-mediated pyroptosis in NASH and the mechanism whereby this occurs, remain unknown. Through our work, we discovered that BBR not only reduces lipid accumulation, alleviates oxidative stress, and inhibits NK-κB signaling and TNF-α expression, but significantly inhibits NLRP3 inflammasome activation and the subsequent pyroptosis. Mechanistically, we further demonstrated that its inhibitory effect on NLRP3 inflammasome and pyroptosis was mediated by the ROS-TXNIP axis (**Figure 8**).

In the current study, we established two distinct-mechanical NASH *in vitro* models by exposing AML12 cells to PA or MCD medium plus LPS and one *in vivo* model by feeding MCD diet to C57BL/6J mouse for 5 weeks. PA, accounting for 20–30% of total fatty acids in the human body (Carta et al., 2017) and the cause of lipotoxicity in hepatocytes, is widely used to establish NASH *in vitro* models (Ji et al., 2018). Deprivation of methionine and choline is known to cause many disturbances in hepatic lipid metabolism due to the decreased availability of methionine and choline for phospholipid synthesis (Rinella et al., 2008; Pickens et al., 2009). Furthermore, LPS, which plays a key role in the pathogenesis of NASH as one of the multi-hits, can cause inflammation in cells. Our results showed that significant lipid accumulation, oxidative stress, and activation of inflammation-associated signaling were observed in the *in vitro* models, and histological features such as lipid accumulation, inflammatory infiltration, and liver injury were occurred in the *in vivo* model, facilitating the investigation performed to determine the effect and molecular mechanism of BBR on NASH.

Recently, increasing evidence have suggested a critical role of GSDMD-mediated pyroptosis in liver diseases (Kofahi et al., 2016; Beier and Banales, 2018; Khanova et al., 2018). Xu et al. demonstrated that GSDMD-N correlates with NAS and fibrosis in NASH patients. Mechanically, GSDMD-N promoted NASH development by mediating hepatic cytokine secretion, macrophage infiltration, NF-ĸB signaling pathway, and lipogenic gene expression (Xu et al., 2017). In the present study, we revealed that BBR can reduce GSDMD-N both *in vitro* and *in vivo*. Furthermore, to the best of our knowledge, this is the first study to reveal this discovery. The roles of BBR on pyroptosis may vary in different diseases. Li et al. (2017) discovered that BBR significantly increased ATP-induced pyroptosis and increased the release of caspase-1, p10, and IL-1β in macrophages. A research in 2016 (Chu et al., 2016) revealed that BBR could induce pyroptosis in HepG2 cells *via* activating caspase-1.

GSDMD, a substrate of inflammatory caspases, can be cleaved by caspase-1, murine caspase-11, and human caspase-4/5 in different conditions (Shi et al., 2015). In alcoholic hepatitis, Khanova et al. demonstrated that caspase 11/4 and not the canonical caspase-1, cleaved GSDMD and played a pivotal pathogenic role (Khanova et al., 2018). In our study, however, elevation of GSDMD-N was mainly caused by the activation of NLRP3-ASC-caspase-1 inflammasome in the NASH cellular model.

It is well known that activation of the NLRP3 inflammasome in the liver promotes the progression of NASH (Csak et al., 2011). During the development of experimental and clinical NAFLD, the expression of hepatic NLRP3 increases (Csak et al., 2014; Wree et al., 2014a; Wree et al., 2014b; Szabo and Petrasek, 2015). Thus, the development of fatty liver disease in mice deficient in NLRP3 (Nlrp3−/−) or its essential components (Asc−/− and Casp1−/− mice) can be avoided if they consume a high fat or nutrient-deficient diet (Dixon et al., 2012; Wree et al., 2014b). Mridha et al. reported that a pharmacologic blockage of NLRP3 reduces liver inflammation and fibrosis in experimental NASH in mice (Mridha et al., 2017), indicating NLRP3 is a potential target of NASH treatment.

Increasing studies have reported that BBR exhibits an inhibitory effect on NLRP3 inflammasome and is a potential candidate for the treatment of various diseases such as atherosclerosis (Jiang et al., 2017), gouty arthritis (Dinesh and Rasool, 2017), and liver injury (Vivoli et al., 2016). A previous study found that BBR inhibits palmitate-induced NLRP3 inflammasome activation by triggering autophagy in macrophages (Zhou et al., 2017), and in our study, besides macrophages, BBR inhibited NLRP3 inflammasome activation as shown by the decrease in NLRP3 protein levels and caspase-1 activity in MCD/LPS or PA-treated hepatocytes and MCD-fed mice liver.

NLRP3 inflammasome can be activated by many DAMPs and PAMPs, including FFA, LPS, and ROS (Abais et al., 2015); however, the mechanism used by BBR to suppress MCD/LPS or PA-induced NLRP3 inflammasome activation was unclear. Following an increase in ROS, TXNIP dissociates from TRX and binds to NLRP3, leading to NLRP3 activation (Zhou et al., 2010). Owing to this, we hypothesized that BBR may suppress NLRP3 inflammasome activation and subsequent pyroptosis *via* the ROS-TXNIP pathway; this is because ROS was decreased by BBR. Our results revealed that NAC, a ROS scavenger, has a similar effect on NLRP3 inflammasome and pyroptosis to BBR as it suppresses the ROX-TXNIP pathway. Besides, a rescue experiment further confirmed that ROS-TXNIP axis played a key role in the inhibitory effect of BBR on NLRP3 inflammasome and pyroptosis in NASH.

Although two *in vitro* models and one *in vivo* model were employed in our study, more *in vivo* studies using different models are still needed to further confirm the protective effects of BBR on NLRP3 inflammasome and pyroptosis in NASH. MCD diet is widely employed in the NASH animal studies and can induce the histological features of steatohepatitis in human NASH. However, its metabolic context such as obesity and insulin resistance are quite different from human NASH (Ibrahim et al., 2016; Farrell et al., 2019). Since no current model can reproduce all major features of severe human NASH, it is recommended at least two different models such as fast food diet and CDAA/HFD/0.2% cholesterol should be used in the future studies, since no murine model depicts all features of human NASH (Farrell et al., 2019).

Several studies have explored the therapeutic effects of BBR on NASH in murine models (Qiang et al., 2016; He Q. et al., 2017; Zhao et al., 2017; Feng et al., 2018; Luo et al., 2019) and humans (Chang et al., 2016; Wei et al., 2016); Qiang et al. demonstrated that demethyleneberberine attenuated lipid accumulation and inflammatory factors secretion with activation of AMPK and inhibition of oxidative stress in MCD diet feeding mice and db/db mice (Qiang et al., 2016). Feng et al. compared the effects of BBR, curcumin, and lovastatin on NAFLD rats and found that BBR in combination with curcumin exhibited better ameliorative effects on rats with non-alcohol fatty liver than lovastatin (Feng et al., 2018). A meta-analysis included 6ix randomized controlled trials involving 501 patients revealed that BBR has positive efficacy on blood lipids, blood glucose, liver function, insulin resistance, and fatty liver condition of NAFLD patients (Wei et al., 2016). However, these studies were mainly focused on the effects and mechanisms of BBR in the regulation of lipid metabolism and hepatic injury in NAFLD. The roles of BBR in the regulation of NLRP3 inflammasome and pyroptosis in NASH *in vivo* has not been clarified, which needs to be determined in the future studies.

In conclusion, we have provided new molecular insights into the protective mechanism of BBR on NASH through its inhibition of NLRP3 inflammasome and the subsequent pyroptosis, in a ROS-dependent manner. Our findings suggest that BBR is a potential candidate for the treatment of NASH.

## Data Availability Statement

The datasets generated for this study are available on request to the corresponding authors.

## Ethics Statement

The animal study was reviewed and approved by Animal Ethics Committee of the Second Affiliated Hospital of Guangzhou Medical University.

## Author Contributions

WM and DZ performed *in vitro* experiments and generated data. YX: study design, data analysis, and manuscript preparation. JX and LY performed *in vivo* experiments and generated data. GY, ZW, QL, and JL: data analysis and figure preparation. TY, CG, and SL: data analysis and interpretation. YZ: generated idea and study design. HY: generated idea, study design, data analysis, and manuscript writing.

## Funding

This work was supported by grants from the Guangdong Natural Science Funds for Distinguished Young Scholar (no. S2013050014121), Science and Technology Program of Guangzhou (no. 201707010470), the China Postdoctoral Science Foundation (no. 2018M640772), Scientific research projects of Guangzhou Education Bureau (no. 201831831), the National Natural Science Foundation of China (nos. 81372634 and 81600350), and a research project grant from Guangdong Province Office of Education (no. 2015KTSCX117).

## Conflict of Interest

The authors declare that the research was conducted in the absence of any commercial or financial relationships that could be construed as a potential conflict of interest.

## Supplementary Material

Figure S1 Effects of Berberine on cell viability and NLRP3 expression in AML12 cells treated by methionine-choline deficient (MCD)/lipopolysaccharide (LPS). AML12 cells were exposed to MCD/LPS in the presence or absence of berberine at concentrations of 1, 5, 10, 20 μM for 24 h. The protein expressions of NLRP3 are assessed by western blot. Data were expressed as mean ± SD of three independent experiments. *p < 0.05 vs MCD+LPS. MCD, Methionine-choline deficient; LPS, lipopolysaccharide.
