# Supplementary figures and images for: Berberine Inhibits Nod-Like Receptor Family Pyrin Domain Containing 3 Inflammasome Activation and Pyroptosis in Nonalcoholic Steatohepatitis via the ROS/TXNIP Axis
Source: Front Pharmacol. 2020 Mar 3;11:185. doi: 10.3389/fphar.2020.00185 (PMC7063468; doi:10.3389/fphar.2020.00185)

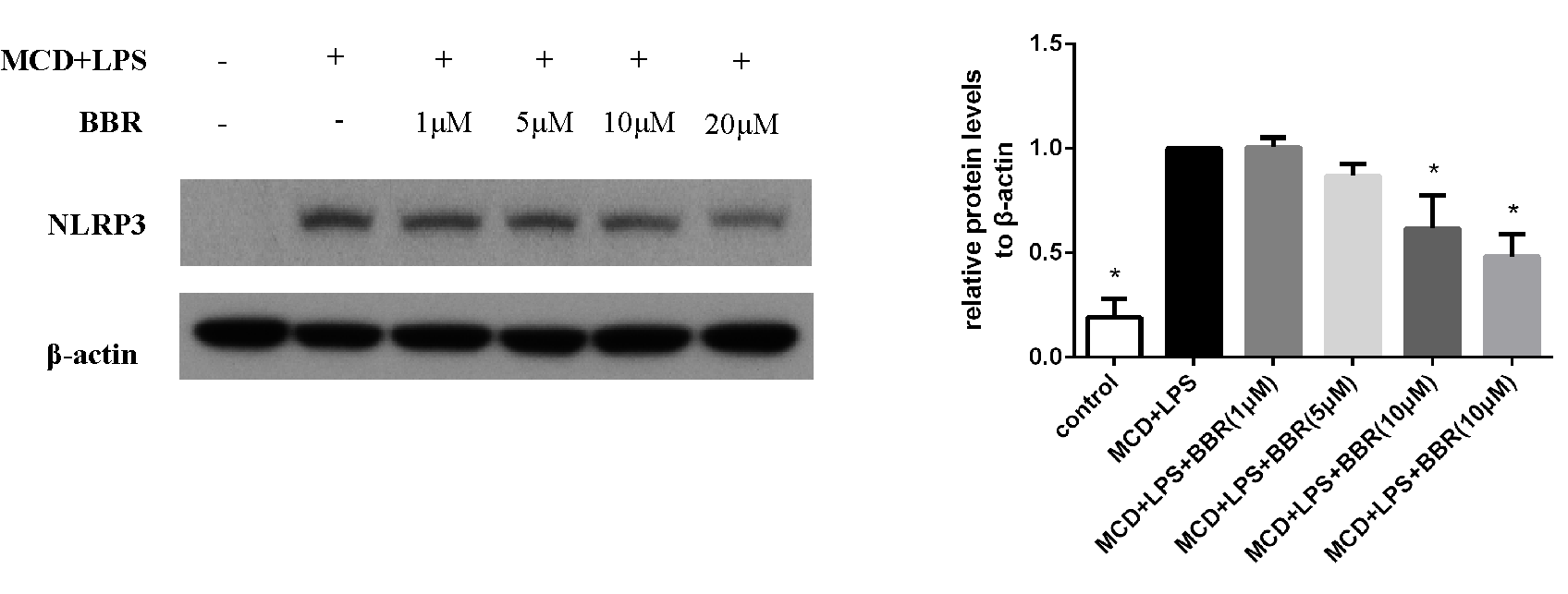

Supplement: Figure S1 — Effects of Berberine on cell viability and NLRP3 expression in AML12 cells treated by methionine-choline deficient (MCD)/lipopolysaccharide (LPS). AML12 cells were exposed to MCD/LPS in the presence or absence of berberine at concentrations of 1, 5, 10, 20 μM for 24 h. The protein expressions of NLRP3 are assessed by western blot. Data were expressed as mean ± SD of three independent experiments. *p < 0.05 vs MCD+LPS. MCD, Methionine-choline deficient; LPS, lipopolysaccharide. [file Image_1.tif]
